# Transcriptomic data of seven flax varieties contrasting in lodging resistance

**DOI:** 10.3389/fpls.2025.1694555

**Published:** 2026-02-06

**Authors:** Daiana A. Krupskaya, Elena N. Pushkova, Valentina A. Krasnova, George S. Krasnov, Tatiana A. Rozhmina, Tatiana Yu. Rybakova, Alexander A. Arkhipov, Nikolai M. Barsukov, Olesya D. Moskalenko, Vera L. Kovalenko, Elizaveta A. Sigova, Ekaterina M. Dvorianinova, Elena V. Borkhert, Alexey A. Dmitriev, Nataliya V. Melnikova

**Affiliations:** 1 Engelhardt Institute of Molecular Biology, Russian Academy of Sciences, Moscow, Russia; 2 National Research University Higher School of Economics, Moscow, Russia; 3 Federal Research Center for Bast Fiber Crops, Torzhok, Russia; 4 Russian State Agrarian University – Moscow Timiryazev Agricultural Academy, Moscow, Russia; 5 Moscow Institute of Physics and Technology, Moscow, Russia

**Keywords:** fiber flax, *Linum usitatissimum*, lodging resistance, transcriptomic data, gene expression, DNA polymorphism

## 1 Introduction

Flax (*Linum usitatissimum* L.) is a versatile crop grown worldwide. Its seeds contain valuable oil, while its stems provide natural fiber. Flax products have a wide range of applications. Flax seeds, in particular, are used in the food industry due to their high content of omega-3 fatty acids, easily digestible proteins, lignans, and fiber. Incorporating flax products into a diet can positively impact human health by promoting digestion and strengthening the immune system (Čeh et al., 2020; Jahan et al., 2024). Linseed oil, derived from flax seeds, has numerous applications in pharmaceuticals and medicine, as well as various industries. It is used in the production of soaps, oilcloth, patent leather, waterproof textiles, paints, varnishes, linoleum, and printing ink. Additionally, it serves as mild insect repellents and antifungal agent (Chaudhary et al., 2016; Hall et al., 2016; Emara et al., 2024). Furthermore, flax fiber is in high demand in modern industries. Its versatility and strength make it an important material for various products. It is used in the production of high-quality textiles, powders, and composite materials (Asyraf et al., 2022; More, 2022).

Biotic (pathogens and diseases) and abiotic environmental factors (heat, drought, salinity, lodging, and waterlogging) significantly reduce flax productivity (Vera et al., 2012; Heller and Byczyńska, 2015; Dubey et al., 2020; Moyse et al., 2023). Therefore, developing new resistant varieties is a top priority in agriculture. Using molecular markers and genome editing to select promising plants can greatly increase breeding efficiency (Zhernova et al., 2025b), but there are currently few informative molecular markers available, particularly for complex traits like lodging resistance.

Flax often suffers from lodging, especially in conditions of heavy precipitation and wind. Mature plants with upper shoots heavily loaded with capsules and numerous branches are especially susceptible (Vera et al., 2012). Lodging is a condition in which flax plants lie flat on the ground due to extreme weather or mechanical stress, making them vulnerable to rot and difficult to harvest with machines. Lodging resistance is a complex multifactorial trait that is influenced by both genetic factors and environmental conditions. Lodging resistance refers to the ability of plants to recover from the stress after exposure to adverse conditions. This includes regaining an upright position. There are ways to reduce lodging in flax plants by selecting the appropriate sowing density and optimal amount of fertilizer (Dey et al., 2022; Xu et al., 2022). Sowing density has been shown to affect lodging resistance by regulating lignin synthesis, but the mechanism varies between highly lodging-resistant and susceptible varieties (Gao et al., 2018; Xu et al., 2022). Phloem fibers and their cell wall characteristics have been shown to play a crucial role in plant lodging resistance (Bourmaud et al., 2015; Ibragimova et al., 2017). Numerous experiments have been conducted in artificially induced lodging conditions - gravistimulation. These experiments have yielded extensive data on the transcriptomic profiles and the microscopic structure of plant organs involved in the response to lodging (Gorshkov et al., 2017; Mokshina et al., 2018, 2024; Gorshkova et al., 2025). It has been demonstrated that other plant tissues contribute to flax resistance to lodging (Petrova et al., 2024). However, information on the genetic basis of this resistance is still extremely scarce. It has been shown that the Lu2560 marker is associated with lodging resistance, explaining 8.9% of the phenotypic variation (Soto-Cerda et al., 2014; You and Cloutier, 2020). Clearly, further research into the flax genome is necessary to identify the molecular mechanisms behind lodging resistance. The aim of this study was to obtain transcriptomic data at various stages of plant development and for various organs for seven flax varieties differing in lodging resistance (five resistant to lodging and two susceptible). Transcriptomic data will help to understand the functions of genes and, therefore, make it possible to use methods of marker-assisted selection and genome editing in flax breeding.

## 2 Material and methods

### 2.1 Plant material

The study included seven flax varieties selected for their differences in lodging resistance. The seeds were obtained from the collection of the Institute for Flax in Torzhok (Russia). Materials for the study were collected from the fields of the Institute for Flax in 2024 at various stages of plant development. The analysis included five lodging-resistant varieties: Rosinka (Ros), Belinka (Bel), Alizee (Ali), Grant (Gr), v-8744-10 (v87); and two lodging-susceptible: k-470 Porkhovsky kryazh (k47) and Priziv 81 (Pri). The flax varieties presented in the study also differ in terms of maturity rate and fiber quality. The early-maturing genotypes include v-8744-10, k-470 Porkhovsky kryazh, and Priziv 81. The late-maturing varieties are Rosinka, Belinka, and Alizee. The medium-ripening genotype is Grant. In terms of spinning ability, Priziv 81 and v-8744-10 have the highest fiber quality, Rosinka and k-470 Porkhovsky kryazh are average, while Belinka, Alizee, and Grant are unsatisfactory. The spinning ability directly depends on the calculated relative breaking strength of the yarn. Rosinka, Alizee, and Grant have a high fiber number, while Belinka, k-470 Porkhovsky kryazh, and Priziv 81 have a medium fiber number, and v-8744-10, on the other hand, has a low fiber number. The fiber number depends on a set of characteristics, including strength, flexibility, fiber length, etc (Rozhmina et al., 2024). We selected a diverse range of flax varieties for this study, as our goal was to obtain a useful and informative set of transcriptomic data that could be used to comprehensively analyze the genetic basis of agriculturally important traits.

**Figure 1:**
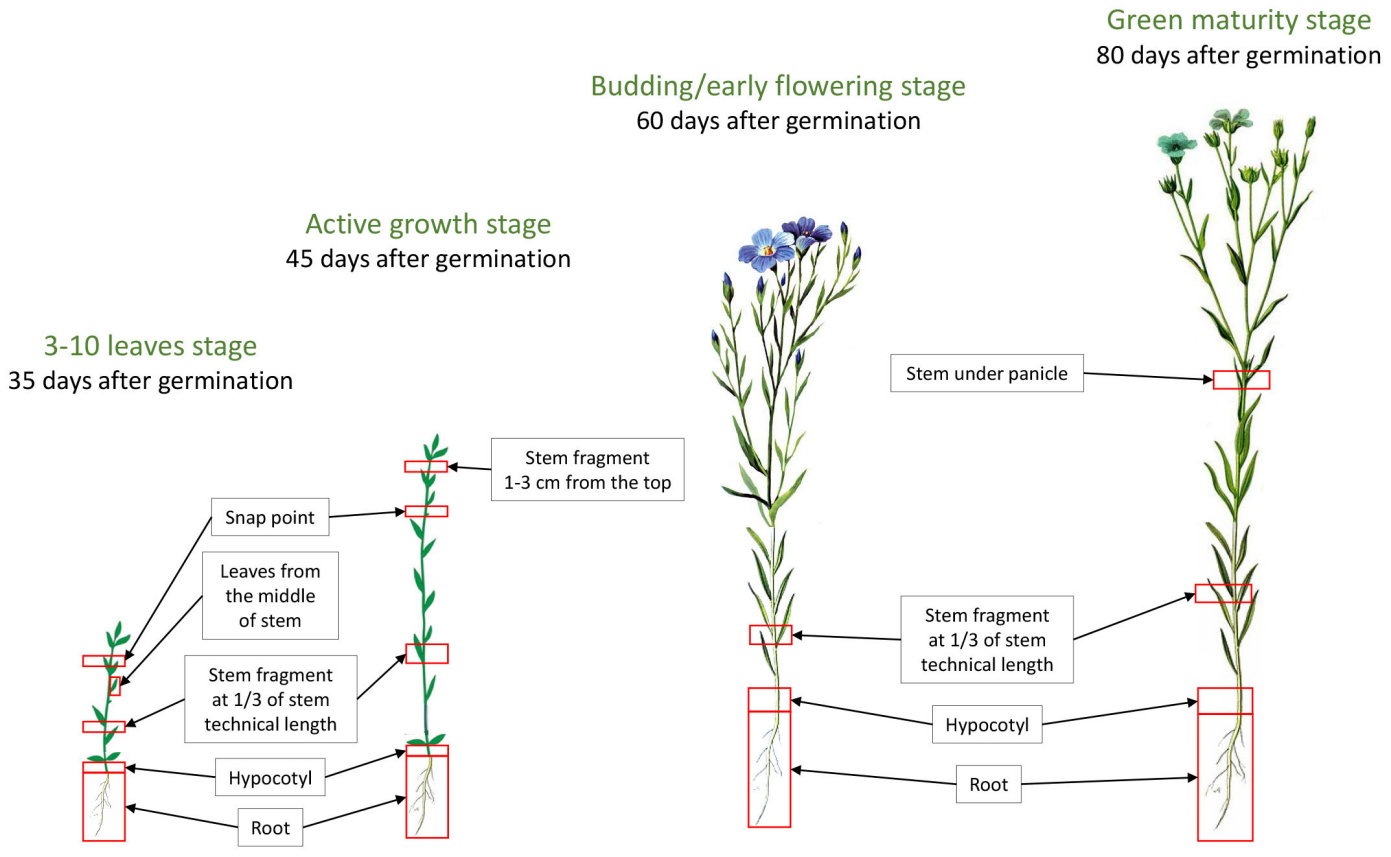
Sample collection at four key stages of flax plant development.

Flax was grown in open field conditions from the end of May to the middle of August, with a density of 2.5 cm per hole, and was additionally watered manually if there was insufficient rainfall. The field experiment was carried out in accordance with the guidelines for flax (Ponazhev et al., 2014). The soil in the experimental area is a sod-medium podzolic loam with a high phosphorus content of 214–350 mg/kg. It is slightly acidic with a pH of 5.4–5.8. Fertilizers and herbicides were not used for this experiment. Samples were collected at four stages of flax development: 1st stage – 3–10 leaves stage (35 days after germination), 2nd stage – active growth stage (45 days after germination), 3rd stage – budding/early flowering stage (60 days after germination), and 4th stage – green maturity stage (80 days after germination). Transcriptomic data were obtained for roots (root), hypocotyls (hyp), stem fragments sampled at 1/3 of stem technical length (stem), snap points (sp), stem fragments 1–3 cm below the apex (stem_1_3_top), stems under panicles (st_panicle), and leaves from the middle third of stems (leaves) (**Figure 1**). The snap point is the point below which the elongation of bast fiber cells has been fully completed. The snap point is present until the end of the linear growth of the stem (Gorshkova et al., 2003). These materials were chosen for the study because the stem and phloem fibers play a crucial role in lodging resistance. It was necessary to collect various parts of the stem, including the point where fibers reach maturity, as well as the root, which anchors the plant in the substrate and contributes to plant lodging resistance. Leaves were collected at the first stage of development as control samples with predictable expression patterns. Each sample was collected in three biological replicates and mixed during RNA extraction, then stored at -70°C. The raw data file names are derived from the genotype abbreviation, developmental stage, and organ being studied, for example, Ros_2_root represents the root of flax variety Rosinka during active growth stage.

### 2.2 RNA isolation

Samples were ground using a disposable pestle inserted into a DeWALT DCD701D2 (DeWALT, Towson, MD, USA) or Makita DF332DZ (Makita, Anjo, Japan) cordless driver drills, at 1200–1500 rpm in 1.5 ml tubes in liquid nitrogen to create a fine powder. During this process, the samples were not allowed to thaw. RNA isolation was performed using the HiPure HP Plant RNA Mini Kit (Magen, Guangzhou, China) with minor modifications to the kit’s instructions, which offer two different lysis buffers for materials of varying complexity. For our study, both buffers were mixed in a 1:1 ratio. The quality of the resulting RNA was assessed by 2100 Bioanalyzer (Agilent Technologies, Santa Clara, CA, USA) and horizontal electrophoresis on a 2% agarose gel, and concentration was measured using a Qubit 4.0 fluorometer (Thermo Fisher Scientific, Waltham, MA, USA).

### 2.3 cDNA library preparation and sequencing on the Illumina platform

The cDNA libraries were prepared using the MagicPure mRNA Kit (TransGen, Beijing, China), TransNGS Fast RNA-Seq Library Prep Kit for Illumina (TransGen), and MagicPure Size Selection DNA beads (TransGen). The quality of the obtained cDNA libraries (correspondence between the length of the obtained libraries and the expected one and the absence of adapter dimers) was assessed on the Qsep1-Plus capillary electrophoresis system (BiOptic, New Taipei City, Taiwan) and by horizontal electrophoresis on a 2% agarose gel. Concentration was evaluated on a Qubit 4.0 fluorometer (Thermo Fisher Scientific). The cDNA libraries were mixed equimolarly and sequenced on a Novaseq X Plus instrument (Illumina, San Diego, CA, USA) in 150 + 150 nucleotide format.

## 3 Description of the obtained transcriptomic data

### 3.1 RNA-seq data characteristics

According to our goal, we have collected a large amount of transcriptomic data from various organs of seven different flax varieties at different stages of development. On average, 9 million reads were obtained per sample: most of the samples (117 out of 119) yielded 5.0-14.5 million reads. Only two samples (Lin_Ali_3_stem and Lin_Pri_3_stem) had about 10 thousand reads each. The *L. usitatissimum* genome (line 3896, NCBI Genome GCA_030674075.2 (Dvorianinova et al., 2023; Zhernova et al., 2025a)) mapping ratio showed substantial variation, mainly depending on the growth stage (on average 95%, 95%, 92%, and 78% for stages 1-4, respectively). Tissue type had a lesser impact on the results of mapping. It should be noted that the invasion of fungal and bacterial pathogens can occur in flax plants, especially at later stages of plant development. The average GC content of the obtained reads was significantly higher in a half of the stage 4 samples (52% compared to 48% in the remaining samples), suggesting the presence of pathogen contamination in these samples.

**Figure 2:**
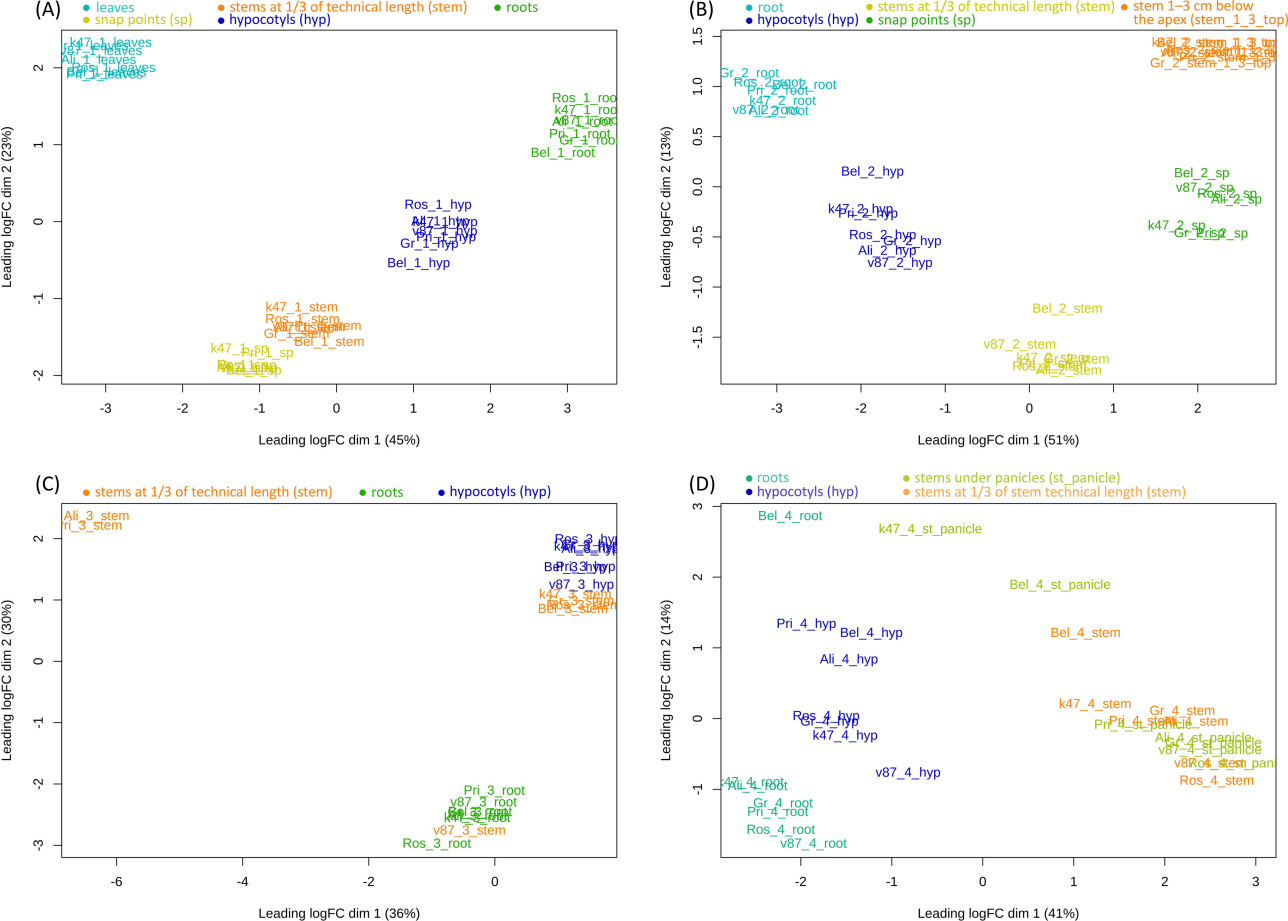
MDS plots (dim1 and dim2) for five resistant and two susceptible to lodging flax varieties at four ontogenetic stages for roots, leaves, and parts of stems, top 2000 genes: **(A)** – 1st stage – 3–10 leaves stage, **(B)** – 2nd stage – active growth stage, **(C)** – 3rd stage – budding/early flowering stage, **(D)** – 4th stage – green maturity stage. Colors indicate different organs.

The residual rRNA content in all samples was below 3.5%, with a mean of 0.7%. The median rRNA content was 0.44% (IQR: 0.20-0.38%). Only 10 out of 119 samples (8.4%) had rRNA levels above 2%. This suggests that the polyA extraction was robust. There was moderate variation in 3’-bias across all 119 samples, with skewness values ranging from 0.35 to 0.80 (median: 0.52, IQR: 0.38-0.48). Despite this variability, there was no discernible effect of the 3’-bias on expression levels, as no tendency toward grouping of samples based on 3’-bias was observed in the MDS plots (**Figure 2**).

### 3.2 Preliminary data analysis

We conducted a preliminary analysis of the obtained data in several different directions to demonstrate the usefulness of the presented data for a variety of purposes. The preliminary analysis was performed using bioinformatics tools, which are detailed in the **Supplementary File S1**.

Preliminary analysis of the data includes expression levels of all genes across various organs, four developmental stages, and seven genotypes of *L. usitatissimum* as read counts per million reads (**Supplementary Table S1**, deposited at https://zenodo.org/records/16859032), analysis of pathogenic contamination of samples (**Supplementary Table S2**), DNA polymorphisms in gene coding regions (**Supplementary Table S3**), and Gene Ontology (GO) enrichment analysis using Overrepresentation Analysis (ORA) for comparisons between lodging-resistant and lodging-susceptible flax genotypes for hypocotyls and stem fragments sampled at 1/3 of stem technical length (**Supplementary Table S4**), as well as visualization of gene expression using a heatmap (**Supplementary Figure S1**) and MDS-plots (**Figure 2**) (in **Figure 2** and **Supplementary Figure S1** we did not use the prefix “Lin_” in the names of the samples for conciseness). From **Figure 2**, it can be seen that samples from the first and the second developmental stages demonstrate clear and robust clustering patterns according to the organ type. Moreover, the stem fragments at 1/3 of stem technical length and the snap points at the first stage are close to each other on the plot, since both groups of samples are parts of the stem, still equally immature at this stage of ontogenesis. During the second growth stage, which is characterized by active plant development and fiber initiation, the MDS plot shows significant differentiation among different stem regions. At the third and fourth stages, infection with fungal pathogens appears. At late stages, due to the damage caused by these pathogens to plant tissues, the transcriptomic profiles become significantly altered. At the 4th stage, samples tend to cluster more based on pathogen infection levels rather than plant tissue type. The most noticeable levels of fungal pathogens were observed in Lin_Bel_4_root and Lin_Bel_4_hyp samples (**Supplementary Table S2**). For samples Lin_Ali_3_stem and Lin_Pri_3_stem, extremely few reads were obtained (about 10 thousand reads), so they also did not fit into the overall picture.

In general, there was a noticeable tendency toward clustering of identical organs among themselves and also a tend to an increase in the number of fungal-contaminated samples as the plant age increased. At the third and fourth stages, the expression profiles in different parts of the stem gradually become more similar. The fourth stage is characterized by a complete mixing of stem fragments at 1/3 of stem technical length and stems under the panicles.

The gene expression heatmap (**Supplementary Figure S1**) shows many distinct clusters of samples corresponding to identical organs, especially at stages 1 and 2 of plant development. This confirms the high quality of the data. Additionally, there are some mixed clusters of close samples, such as those of snap points and stem fragments 1–3 cm from the top, as well as stems under the panicles and stem fragments at 1/3 of stem technical length. The transcriptomic data we obtained allow scientists to significantly enhance understanding of flax gene expression in various organs at different stages of ontogenesis. Our research included flax varieties with different levels of resistance to lodging. Under field conditions without lodging, it can be defined which varieties are more likely to be affected by lodging based on indirect morphological features, such as the curvature of the stem base, stem thickness, and internode length. However, the identification of resistant and susceptible flax varieties in the field is challenging due to the complex and variable influence of environmental factors. Additionally, experienced experts are required for assessing plant features and determining specific stages of development. Transcriptomic data obtained can be valuable for assessing the involvement of different genes in plant processes, particularly lodging resistance. In addition, expression in organs most vulnerable to lodging can be compared with expression in organs with a high likelihood of not participating in the lodging response. These data allow one to analyze the gene expression separately in resistant and susceptible flax genotypes, compare samples, and search for expression patterns related to key ontogenetic stages.

Among other things, we also presented data on single-nucleotide polymorphisms (SNPs) in gene sequences. Data on polymorphisms provide additional information for the comparative analysis of the studied flax genotypes and assessing differences between certain genes in different genotypes (**Supplementary Table S3**). It is important to note that these studies can be conducted not only for genes associated with lodging but also genes involved in other flax traits, including economically important ones.

In order to show how else the data obtained can be used, we conducted a preliminary Gene Ontology (GO) enrichment analysis using Overrepresentation Analysis (ORA) for comparisons between lodging-resistant and lodging-susceptible flax genotypes (**Supplementary Table S4**). The analysis was performed separately for hypocotyls (hyp) and stem fragments (stem). This analysis revealed statistically significant differences in gene expression between resistant and susceptible genotypes. In **Supplementary Table S4**, each cell represents the overrepresentation score for a specific GO term. The table shows the p-values for GO terms enriched among up- and downregulated genes at all stages of flax development. Red indicates overrepresentation of GO terms by upregulated differentially expressed genes (DEGs) in resistant genotypes, while blue indicates overrepresentation by downregulated DEGs. Mixed colors signify overrepresentation by both up- and downregulated DEGs. Color intensity corresponds to the overrepresentation score, calculated as -log10(p-value) from the hypergeometric test. To visually distinguish the direction of regulation, the scores for downregulated DEGs were multiplied by -1. To enable comparison across different gene list sizes, the analysis was run independently for the top-50, top-100,…, up to top-5000 up- or downregulated DEGs, and a size-based correction factor (ranging from 0.25 to 2.0) was applied to the p-values. The table displays the highest overrepresentation score across all these lists, where a negative score indicates overrepresentation by downregulated DEGs and a positive score by upregulated DEGs. Our data allow one to conduct a similar expression analysis on any combination of samples. This will be useful in identifying the key genes responsible for the formation of agriculturally important traits, and as a result, developing valuable flax varieties.

We managed to obtain a significant amount of transcriptomic data for seven flax varieties. The special value of our data lies in the fact that they are collected from different organs and at four different stages of ontogenesis. Flax genotypes can vary greatly in their mechanisms of resistance to lodging, and therefore, it is important to examine samples of both resistant and susceptible genotypes simultaneously. The diversity of samples presented allows the search for universal patterns and genes that play a crucial role in resistance to lodging at the most critical stages in organs involved in the lodging response. Extensive material of transcriptomic data from different parts of the stem at various stages was obtained. Therefore, these data will be valuable for studying the main genetic determinants of fiber flax traits related to stem and fiber characteristics. It will also be possible to investigate the development of specific parts of the stem in different flax genotypes. The data we obtained allow one not only to compare the expression levels of genes (**Supplementary Table S1**), but also their sequences and the presence of polymorphisms (**Supplementary Table S3**). In other words, it is possible to search for SNPs that can also cause economically important traits. In addition, we presented a preliminary analysis of the differential expression of resistant and susceptible flax varieties in the hypocotyl and stem (**Supplementary Table S4**). The data presented in this study are suitable for a wide range of tasks and further more in-depth analysis and will enable the search for key molecular mechanisms underlying stress resistance and other valuable features, which could facilitate the development of improved flax varieties.

## Data Availability

The datasets presented in this study can be found in online repositories. The names of the repository/repositories and accession number(s) can be found below: https://www.ncbi.nlm.nih.gov/sra/, PRJNA1305415.

## Supplementary material

Supplementary Figure 1 Expression heatmap based on log_2_(CPM/avg.CPM) of all genes in various organs, four developmental stages, and seven flax varieties. The sample names are derived from the genotype abbreviation (Ros - Rosinka, Bel - Belinka, Ali - Alizee, Gr - Grant, v87 - v-8744-10, k47 - k-470 Porkhovsky kryazh, and Pri - Priziv 81), developmental stage (1 – 3–10 leaves stage, 35 days after germination; 2 – active growth stage, 45 days after germination, 3 – budding/early flowering stage, 60 days after germination; 4 – green maturity stage, 80 days after germination), and organ being studied (root - roots, hyp - hypocotyls, stem - stem fragments sampled at 1/3 of stem technical length, sp - snap points, stem_1_3_top - stem fragments 1–3 cm below the apex, st_panicle - stems under panicles, leaves - leaves from the middle third of stems), for example, Ros_2_root represents roots of flax variety Rosinka during plant active growth stage.

Supplementary File 1 Methods for the preliminary analysis of transcriptomic data.

Supplementary Table 1 Expression levels of all genes across various organs, four developmental stages, and seven genotypes of *L. usitatissimum* as read counts per million (CPM). **Supplementary Table S1** is deposited at https://zenodo.org/records/16859032.

Supplementary Table 2 Percentage of RNA-Seq reads of the studied flax samples that mapped to genomes of different pathogens.

Supplementary Table 3 DNA polymorphisms in gene coding regions (CDS) of seven flax varieties based on the analysis of transcriptomic data.

Supplementary Table 4 Summary of the Gene Ontology (GO) enrichment analysis (particularly, overrepresentation analysis, ORA) for genes differentially expressed between lodging-resistant and lodging-susceptible flax varieties for hypocotyls (hyp) and stem fragments sampled at 1/3 of stem technical length (stem).
