# Supplementary figures and images for: Transcriptomic data of seven flax varieties contrasting in lodging resistance
Source: Front Plant Sci. 2026 Feb 6;16:1694555. doi: 10.3389/fpls.2025.1694555 (PMC12920455; doi:10.3389/fpls.2025.1694555)

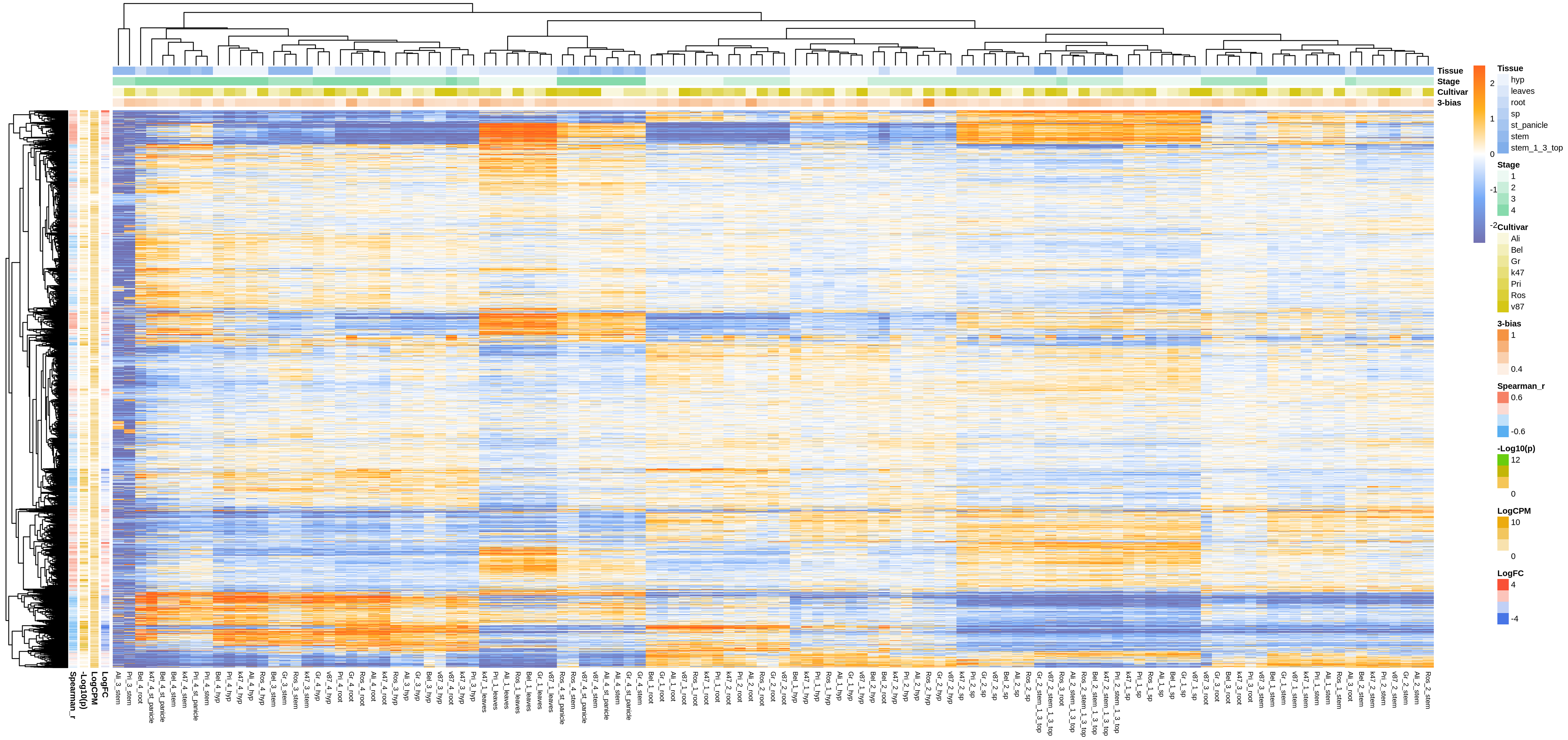

Supplement: Supplementary Figure 1 — Expression heatmap based on log2(CPM/avg.CPM) of all genes in various organs, four developmental stages, and seven flax varieties. The sample names are derived from the genotype abbreviation (Ros - Rosinka, Bel - Belinka, Ali - Alizee, Gr - Grant, v87 - v-8744-10, k47 - k-470 Porkhovsky kryazh, and Pri - Priziv 81), developmental stage (1 – 3–10 leaves stage, 35 days after germination; 2 – active growth stage, 45 days after germination, 3 – budding/early flowering stage, 60 days after germination; 4 – green maturity stage, 80 days after germination), and organ being studied (root - roots, hyp - hypocotyls, stem - stem fragments sampled at 1/3 of stem technical length, sp - snap points, stem_1_3_top - stem fragments 1–3 cm below the apex, st_panicle - stems under panicles, leaves - leaves from the middle third of stems), for example, Ros_2_root represents roots of flax variety Rosinka during plant active growth stage. [file Image1.tif]
