# Supplementary material for: Transcriptomic data of seven flax varieties contrasting in lodging resistance
Source: Front Plant Sci. 2026 Feb 6;16:1694555. doi: 10.3389/fpls.2025.1694555 (PMC12920455; doi:10.3389/fpls.2025.1694555)
Supplement: Supplementary File 1 — Methods for the preliminary analysis of transcriptomic data. [file Supplementaryfile1.docx]

**Supplementary File S1.** Methods for the preliminary analysis of transcriptomic data.

Raw reads were quality-checked with FastQC v0.11.9 (https://www.bioinformatics.babraham.ac.uk/projects/fastqc/) and trimmed using Trimmomatic v0.38 ([Bolger et al., 2014](#_ENREF_1)) with the following parameters: TRAILING:24 SLIDINGWINDOW:4:14 MINLEN:40 and also residual adapters were removed.

To evaluate residual ribosomal RNA content, 100,000 randomly chosen reads per sample were aligned to *L. usitatissimum* rRNA genes (Genbank accession number EU307117) using bowtie2 v2.3.5.1 with the following parameters: --local -D 25 -R 4 -N 1 -L 17 -i S,1,0.50 ([Langmead and Salzberg, 2012](#_ENREF_8)).

The trimmed paired-end reads were mapped to the *L. usitatissimum* genome assembly (line 3896, GCA_030674075.2 ([Dvorianinova et al., 2023](#_ENREF_5))) using STAR (v2.7.3a) ([Dobin et al., 2012](#_ENREF_4)) in one pass mode with parameters: --outFilterMismatchNmax 12 --outFilterMultimapNmax 10 --outSAMstrandField intronMotif --outFilterIntronMotifs RemoveNoncanonicalUnannotated --quantMode TranscriptomeSAM. The maximum number of allowed mismatches (--outFilterMismatchNmax) was increased to 12 from the default value of 10. This adjustment accounts for potential genetic divergence of the cultivated varieties from the reference genome and the considerable read length of 150 nucleotides. Genome annotation ([Zhernova et al., 2025](#_ENREF_14)) GTF file was also supplied to STAR. Subsequently, 3'-bias analysis was performed using RSeQC v2.6.4 ([Wang et al., 2012](#_ENREF_12)). For BED file preparation, we utilized the aforementioned annotation ([Zhernova et al., 2025](#_ENREF_14)) processed with standalone UCSC utilities gtfToGenePred and genePredToBed ([Kuhn et al., 2012](#_ENREF_7)).

Next, we extracted reads unmapped to the *L. usitatissimum* genome, remapped them with bowtie2 (in single-end mode) ([Langmead and Salzberg, 2012](#_ENREF_8)) and extracted unmapped reads once more. These reads were screened for microbial contaminants with Kraken 2 (maxikraken2_1903_140GB database) ([Wood et al., 2019](#_ENREF_13)). However, the database did not contain some key pathogens, and also annotation rate for deep taxonomic levels (e.g. species) was lowered. Hence, we manually analyzed several dozen unmapped reads from various samples with online NCBI BLASTn suite (nr/nt database) ([Camacho et al., 2009](#_ENREF_2)). This revealed hits to fungal genomes (*Septoria linicola* (Speg.) Garass., *Fusarium oxysporum* Schltdl., etc), known plant pathogens, some of which were not found with Kraken.

Next, we downloaded genomes of ~30 most abundantly found or well-known flax pathogens. For each sample, reads unmapped to *L. usitatissimum* were independently mapped to these genomes using bowtie2 (v2.3.5.1) with default parameters ([Langmead and Salzberg, 2012](#_ENREF_8)). Finally, alignment rate was recalculated to the original read count to quantify pathogen load. This approach enabled identification of samples with the highest pathogen contamination levels.

Subsequently, we performed gene expression analysis for flax genes. Read counting per gene was performed with featureCounts v2.0.0 ([Liao et al., 2013](#_ENREF_9)). Differential gene expression was estimated using edgeR 4.0.2 package for R v4.3.2 ([Robinson et al., 2009](#_ENREF_11)). Data normalization was done with TMM method. In the further analysis, we included genes with CPM > 1 for at least 20% of samples. On average, 30 (±2) out of 39 thousand genes passed the filter. Based on the normalized gene expression data, MDS plots and heatmaps were created. Hierarchical clustering was done using the distance matrix calculated as 1-cor (Pearson's correlation coefficient for log2 CPM values). In Figure 2 and Supplementary Figure S1 we did not use the prefix "Lin_" in the names of the samples for conciseness.

The threshold CPM > 1 for at least 20% of samples avoids excluding biologically relevant genes that may only be active in specific subgroups/conditions, yet still removes the majority of noise from genes that are rarely detected. The CPM threshold of 1 is quite common and is calculated based on the library size. As stated in one of the basic papers on differential gene expression analysis (which presents the edgeR package), the exact threshold value is not critically important because the downstream differential expression analysis is not sensitive to small changes in this parameter ([Chen et al., 2016](#_ENREF_3)). The 20% threshold provides a balance: it is stringent enough to remove most spurious/uninformative features but not so stringent that it discards genes with condition-specific expression patterns. This threshold was chosen based on the approximate size of a sample subgroup (e.g., one tissue or stage). However, with this 20% threshold, genes that are selectively expressed strictly at one stage in one tissue might be excluded.

For variant calling, BAM files were processed with GATK SplitNCigarReads 4.1.8.0 ([Poplin et al., 2017](#_ENREF_10)). Marking duplicates was not performed. Then, freebayes 1.3.7 was used for SNP calling (min. total coverage 100, min. PhredQ for SNP 25, min. mapping quality 20, min. base quality 20) (Garrison and Marth, 2012[Garrison and Marth, 2012](#_ENREF_6)). The search was limited to the coding regions fetched from the genome annotation.

Next, we performed a Gene Ontology (GO) enrichment analysis, specifically an Overrepresentation Analysis (ORA). The analysis included differentially expressed genes (DEGs) with a p-value < 0.05 according to the QL F-test. The analysis was carried out using the 'goseq' package and the non-central hypergeometric test (Wallenius test). ORA was conducted independently for various top-DEG lists (top-50, 100, 200, 500, 1000, 2000, 5000), separately for up- and downregulated genes (identified in the comparison between lodging-resistant and lodging-susceptible varieties). As expected, different sizes of gene lists yielded different sets of enriched GO terms. Subsequently, an overrepresentation score was calculated as -log10(p-value) of the hypergeometric test. Since the statistical significance in ORA (i.e., p-values) is highly dependent on the size of the gene list, a correction factor (ranging from 0.25 to 2.0) for the size of the analyzed top-DEG list was incorporated into the score.

**References**

Bolger, A.M., Lohse, M., and Usadel, B. (2014). Trimmomatic: a flexible trimmer for Illumina sequence data. *Bioinformatics* 30(15)**,** 2114-2120. doi: 10.1093/bioinformatics/btu170.

Camacho, C., Coulouris, G., Avagyan, V., Ma, N., Papadopoulos, J., Bealer, K., et al. (2009). BLAST+: architecture and applications. *BMC Bioinformatics* 10(1)**,** 421. doi: 10.1186/1471-2105-10-421.

Chen, Y., Lun, A.T., and Smyth, G.K. (2016). From reads to genes to pathways: differential expression analysis of RNA-Seq experiments using Rsubread and the edgeR quasi-likelihood pipeline. *F1000Research* 5**,** 1438. doi: 10.12688/f1000research.8987.2.

Dobin, A., Davis, C.A., Schlesinger, F., Drenkow, J., Zaleski, C., Jha, S., et al. (2012). STAR: ultrafast universal RNA-seq aligner. *Bioinformatics* 29(1)**,** 15-21. doi: 10.1093/bioinformatics/bts635.

Dvorianinova, E., Pushkova, E., Bolsheva, N., Rozhmina, T., Zhernova, D., Sigova, E., et al. (2023). Improving genome assembly of flax line 3896 with high-precision Illumina reads. *Russian Journal of Genetics* 59(Suppl 2)**,** S237-S240. doi: 10.1134/S102279542314003X.

Garrison, E., and Marth, G. (2012). Haplotype-based variant detection from short-read sequencing. *arXiv preprint arXiv:1207.3907*. doi: 10.48550/arXiv.1207.3907.

Kuhn, R.M., Haussler, D., and Kent, W.J. (2012). The UCSC genome browser and associated tools. *Briefings in Bioinformatics* 14(2)**,** 144-161. doi: 10.1093/bib/bbs038.

Langmead, B., and Salzberg, S.L. (2012). Fast gapped-read alignment with Bowtie 2. *Nature Methods* 9(4)**,** 357-359. doi: 10.1038/nmeth.1923.

Liao, Y., Smyth, G.K., and Shi, W. (2013). featureCounts: an efficient general purpose program for assigning sequence reads to genomic features. *Bioinformatics* 30(7)**,** 923-930. doi: 10.1093/bioinformatics/btt656.

Poplin, R., Ruano-Rubio, V., DePristo, M.A., Fennell, T.J., Carneiro, M.O., Van der Auwera, G.A., et al. (2017). Scaling accurate genetic variant discovery to tens of thousands of samples. *biorxiv***,** 201178. doi: 10.1101/201178.

Robinson, M.D., McCarthy, D.J., and Smyth, G.K. (2009). edgeR: a Bioconductor package for differential expression analysis of digital gene expression data. *Bioinformatics* 26(1)**,** 139-140. doi: 10.1093/bioinformatics/btp616.

Wang, L., Wang, S., and Li, W. (2012). RSeQC: quality control of RNA-seq experiments. *Bioinformatics* 28(16)**,** 2184-2185. doi: 10.1093/bioinformatics/bts356.

Wood, D.E., Lu, J., and Langmead, B. (2019). Improved metagenomic analysis with Kraken 2. *Genome Biology* 20(1)**,** 257. doi: 10.1186/s13059-019-1891-0.

Zhernova, D.A., Arkhipov, A.A., Rozhmina, T.A., Zhuchenko, A.A., Bolsheva, N.L., Sigova, E.A., et al. (2025). Transcriptome map and genome annotation of flax line 3896. *Frontiers in Plant Science* 16**,** 1520832. doi: 10.3389/fpls.2025.1520832.
